# Influence of Age on Treatment and Prognosis in Ovarian Cancer Patients

**DOI:** 10.3390/cancers17091397

**Published:** 2025-04-22

**Authors:** Gemma Mancebo, Josep Maria Sole-Sedeno, Berta Fabregó, Giovanna Pinto, Adrián Vizoso, Marta Alvarez, Rosa Ana Sabaté-Garcia, Ester Miralpeix

**Affiliations:** 1 Department of Obstetrics and Gynecology, Hospital del Mar, 08003 Barcelona, Spain; jsole@hmar.cat (J.M.S.-S.); bfabrego@hmar.cat (B.F.); malvarezduran@hmar.cat (M.A.); emiralpeix@hmar.cat (E.M.); 2 Faculty of Medicine and Life Science, Pompeu Fabra University, 08003 Barcelona, Spain; 3 Department of Epidemiology, Hospital del Mar, 08003 Barcelona, Spain; avizoso@researchmar.net; 4 Department of Geriatrician, Hospital del Mar, 08003 Barcelona, Spain; rsabate@hmar.cat

**Keywords:** ovarian neoplasms, aged, prognosis, disease-free survival

## Abstract

**Background**: Ovarian cancer, particularly in advanced stages, requires cytoreductive surgery followed by chemotherapy. A significant proportion of patients are elderly, yet older women are often treated with non-standard regimens despite a lack of consistent evidence linking age to prognosis. The aim of this study is to assess age-specific differences in treatment and survival outcomes for ovarian cancer in women aged 70 years or older. **Methods**: This retrospective study included ovarian cancer patients treated at the Hospital del Mar, Barcelona, between 2016 and 2022. Patients were stratified into two groups: <70 and ≥70 years. Clinical and pathological data were analyzed, and hazard ratios (HR) for overall survival (OS) and disease-free survival (DFS) were calculated using Cox proportional hazards regression models. Multivariate analysis was performed to compare outcomes. **Results**: A total of 110 patients were included (73 <70 years, 37 ≥70 years). Among the older group, 80.5% were diagnosed at advanced stages (III–IV), compared to 63% in the younger group (*p* = 0.012). Patients aged ≥70 were more likely to undergo interval surgery (*p* = 0.053) and receive non-standard treatment (*p* = 0.023). Complete cytoreduction was achieved in 95.8% of younger patients versus 81.3% of older patients (*p* = 0.024). Age ≥70 did not significantly impact DFS (*p* = 0.091), but OS was significantly worse in the older group (44.4% vs. 67.2%, *p* = 0.014). **Conclusions**: Older women (≥70 years) with ovarian cancer are more likely to be diagnosed at advanced stages, receive non-standard treatment, and achieve suboptimal cytoreduction compared to younger patients. While DFS was similar across age groups, older age was associated with worse OS, highlighting the need for age-tailored treatment strategies.

## Simple Summary

Ovarian cancer treatment typically involves surgery and chemotherapy, but older women often receive non-standard therapies despite limited evidence linking age to prognosis. In this study, we analyzed data from patients treated at the Hospital del Mar to understand how age influences treatment decisions and survival outcomes. We found that women aged 70 or older were more frequently diagnosed at advanced stages, received different treatment approaches, and had lower rates of complete tumor removal compared to younger patients. While disease-free survival was similar between age groups, overall survival was worse in older women. These findings highlight the need for treatment strategies that consider the specific needs of elderly patients to improve their outcomes.

## 1. Introduction

Ovarian cancer is a heterogeneous disease that stands as the second most common gynecological tumor, with an incidence of 11.8 per 100,000 women [1,2]. The risk of developing the disease increases with age, with over half occurring in patients aged 65 or older [3,4]. As it often presents with non-specific symptoms, up to 70% of cases are diagnosed in advanced stages [1,5]. Consequently, ovarian cancer has the highest mortality rate among gynecological cancers, as this late-stage diagnosis significantly hinders effective treatment [5,6].

The standard treatment for advanced-stage ovarian cancer usually consists of complete cytoreductive surgery followed by six cycles of adjuvant chemotherapy, with platinum and taxane-based drugs [1,5]. Complete cytoreduction means total resection of any evidence of macroscopic intra-abdominal tumor, which often implies extended surgery with retroperitoneal, digestive, urological and peritoneal resections [5,7]. Primary surgery for ovarian cancer is considered the gold-standard treatment, with a 50% survival rate at 5 years when complete cytoreduction is accomplished [8]. Patient selection for primary surgery mainly considers the patient’s basal physical state and tumoral burden. However, especially in older patients, frailty and overall health can define surgical comorbidity and determine the choice of alternative treatments [6,9]. When complete cytoreduction is deemed unattainable or too risky based on the patient’s condition, neoadjuvant chemotherapy is administered prior to interval surgery [5]. Studies like EORTC and CHORUS have demonstrated the effectiveness of neoadjuvant chemotherapy in selected cases initially deemed non-cytoreductive [10,11,12]. Therefore, personalized treatments are essential to ensure each patient receives the optimal care, maximizing both survival rates and quality of life.

The hypothesis of the study is that elderly ovarian cancer patients undergo non-standard treatment more often compared to younger patients. The study aimed to categorize ovarian cancer in older women in terms of tumor characteristics, treatment approaches, and oncological outcomes and assess age-specific survival differences, considering overall survival (OS) and disease-free survival (DFS).

## 2. Materials and Methods

### 2.1. Study Design and Participants

This single institution, observational longitudinal retrospective study was conducted in the Hospital del Mar, Barcelona, between January 2016 and December 2022. Women newly diagnosed of ovarian cancer with medical registers and necessary data available were included.

### 2.2. Data Collection and Outcomes

Demographic, clinical, tumor-related, and treatment-related data were obtained from the electronic medical records from the Hospital del Mar. For the purpose of the study, patients were categorized into two groups based on their age at diagnosis: <70 or ≥70 years old, following geriatric recommendations about screening frailty on older age [13]. To analyze patient comorbidity, the Charlson Index was calculated using an online calculator from SAMIUC (Sociedad Andaluza de Medicina Intensiva y Unidades Coronarias) [14]. Tumor stage was defined according to the 2014 International Federation of Gynecology and Obstetrics (FIGO) staging system [15]. The type of surgery was categorized as “primary” when cytoreductive surgery was performed before adjuvant chemotherapy or if no chemotherapy was administered, “interval” when surgery was performed after two or four cycles of neoadjuvant chemotherapy and after surgery chemotherapy continued to complete six cycles, and “rescue” when surgery was performed after six cycles of neoadjuvant chemotherapy. Additionally, based on residual macroscopic tumor, surgery was considered complete (R0) and incomplete (R1) cytoreduction [7]. In summary, treatment approaches were classified into two categories: “standard” treatment, which included primary or interval debulking surgery. “Non-standard” treatment encompassed any therapeutic strategy that did not involve cytoreductive surgery, such as exclusive chemotherapy, palliative surgery, or other non-curative interventions. Maintenance treatment was defined as any type of treatment administered after the finalization of the main treatment, considering tumor characteristics, preferences, age-specific toxicity rates, and patient’s needs according to oncological guidelines including PARP Inhibitors or Antiangiogenic Therapy [16]. To analyze the complexity of the surgeries performed, the Aletti Surgical Complexity Scoring System was used, and the score obtained was divided into three groups: low risk (≤3), intermediate risk (4–7), and high risk (≥8) [17]. Regarding surgery complications, the 2004 Clavien Dindo classification was used [18]. The follow-up period was defined as the time between first treatment and disease’s recurrence (DFS), any cause of death (OS), or last medical appointment.

### 2.3. Ethical Considerations

The study was approved by the ethics committee of the Hospital del Mar number CEIm 2023/11249 and was carried out in compliance with the guidelines of the Declaration of Helsinki, Fortaleza, Brazil, 2013. A waiver of informed consent was obtained from the institutional ethical review board from the home institution.

### 2.4. Statistical Analysis

The variables were described with relative and absolute frequencies for categorical variables, and mean, quartiles or standard deviation for continuous variables. A chi-square test was used to study differences between groups for categorical variables. Fisher’s exact test was used when the frequency of any cell in the contingency table was lower than five. For continuous variables, the non-parametric Kruskal–Wallis test was used. Overall survival (OS) and disease-free survival (DFS) were studied using survival analysis with Kaplan–Meier curves and log-rank tests to determine differences between age groups. Univariate Cox proportional hazards regression models were used to identify potential associated variables. Multivariate analysis was conducted using a Cox proportional hazard regression model, considering age as the primary factor. Other significant variables, identified through univariate analysis and based on their clinical relevance, were included as covariates in the model. Results were expressed as hazard ratios (HR) with corresponding 95% confidence intervals. All *p*-values lower than the significance level of 0.05 were considered significant. Analyses were performed in R version 4.2.2.

## 3. Results

During the study period, a total of 110 patients were included with a mean age of 62.9 (SD ± 14.03) years at time of diagnosis. Of the total of patients included, 73 (66.4%) were younger than 70 years old and 37 (33.6%) were 70 or older. Compared with patients under 70, older patients had a higher Comorbidity Charlson Index (*p* < 0.001) and a higher ASA score (*p* = 0.013). Concerning tumoral characteristics, 80.5% of older patients were diagnosed at advanced stages (III–IV), compared to 63% in <70 patients (*p* = 0.012). There were no differences when considering histological type of tumor or presence of tumoral ascites at the time of diagnosis (Table 1).

Regarding treatment type, 80.9% of patients of our cohort received standard treatment while 19.1% received non-standard treatment. Patients ≥70 were more likely to receive non-standard treatment compared to younger patients (32.4% vs. 12.3%. *p* = 0.023). Out of all patients who underwent surgery, 89.3% received either primary or interval surgery. When comparing by age, 93.0% of patients under 70 underwent primary or interval surgery, while this rate was 81.2% among older patients. Specifically, older patients were more likely to undergo interval surgery (31.2%) as well as rescue surgery (18.8%) compared to patients under 70 (19.7% and 7%, consecutively, *p* = 0.053). Complete cytoreduction was achieved in 95.8% of patients <70 compared to 81.3% in older patients (*p* = 0.024). However, when considering the Aletti Score, duration of surgery, and surgery complications (Clavien Dindo Classification), there were no significant differences when comparing age groups. Regarding chemotherapy variables, 80% of patients of our cohort received chemotherapy, without differences when considering chemotherapy administration, completeness of cycles, or chemotherapy complications between age groups. At the time of the last follow-up, 75 (68.2%) patients included were alive; particularly, 56 (76.7%) patients under 70 years old compared to 19 (51.4%) patients older (*p* = 0.006). After a median overall follow-up of 21.6 months (IQR: 13.8–32.5), 54 out of 110 patients (56.3%) experienced relapse. Among patients aged 70 years or older, 20 (71.4%) relapsed, compared to 34 (50%) of those younger than 70 (*p* = 0.054) (Table 2).

**Figure 1:**
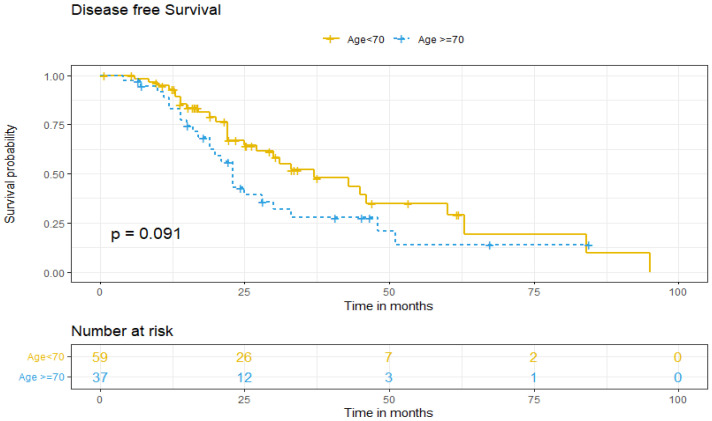
Disease-free survival according to age group.

Regarding DFS between both age groups, five-year DFS was 13% for older patients and 37% for patients under 70 (*p* = 0.091) (Figure 1). DFS was adjusted for patient-related factors that could potentially affect survival. Univariate and multivariate analysis showed that age ≥70 (aHR = 1.24. 95% IC, 0.55–2.78, *p* > 0.05) and non-standard treatment (aHR = 0.58. 95% IC, 0.24–1.4, *p* > 0.05) did not independently predict worse DFS of ovarian cancer patients. However, being diagnosed at higher stages (III–IV) increases the risk of recurrence by more than three times (aHR 3.44; 95% IC, 1.50–7.87, *p* = 0.003), and the presence of tumoral ascites doubles it (aHR 2.23; 95% IC, 1.15–4.35, *p* = 0.018), as independent predictive factors (Table 3).

**Figure 2:**
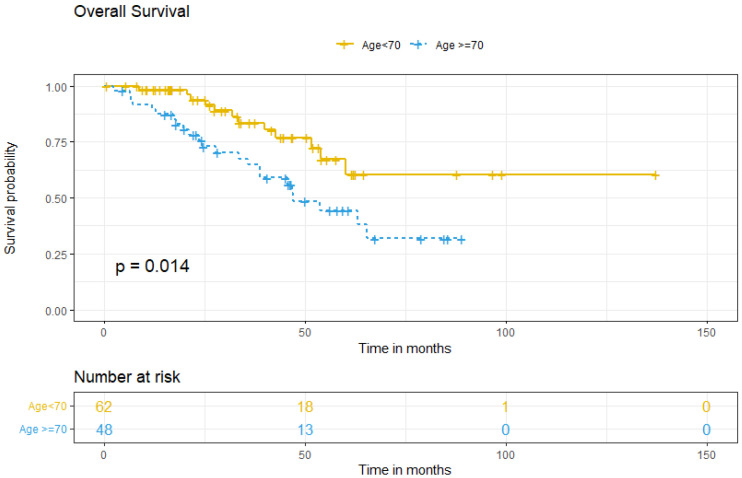
Overall survival according to age group.

Survival analysis between groups showed that the five-year OS for patients <70 was 67.2%, compared to 44.4% for older patients (*p* = 0.014) (Figure 2). Multivariate analysis for OS showed that ≥70, comorbidity, FIGO stage at diagnosis, tumoral ascites, and treatment type did not independently predict worse OS (Table 4).

## 4. Discussion

To our knowledge, this study represents one of the few investigations that assesses specific differences among ovarian cancer patients in terms of age. Our study showed that older patients diagnosed with ovarian cancer are less likely to receive standard treatment in the form of surgery (primary or interval) plus chemotherapy, which could explain their worse OS when compared to younger patients. However, there is no consistent evidence evaluating the impact of older age in survival for ovarian cancer [3,9].

Among ovarian cancer patients, there is a significant portion of elderly women, of whom treatment is controversial [3,4]. Some studies indicate that older patients with ovarian cancer tend to receive less aggressive treatment due to concerns about increased surgical risks and comorbidities, even though some guidelines consider frailty as eligibility criteria to neoadjuvant chemotherapy [9]. In this line, recent studies suggest that older patients could benefit from geriatric screening tests, with the goal of identifying patients with age-related problems not typically identified by a routine history and physical examination, like nutritional issues or functional impairments, who could benefit from geriatric interventions and optimization programs, such as prehabilitation, and on the other hand, can help select an appropriate cancer treatment [13,19]. Therefore, relying solely on age to exclude patients from standard treatment might overlook many individuals, potentially impacting their prognosis. Hence, it’s crucial to investigate whether older patients are indeed more frequently excluded from standard treatment and whether OS and DFS vary by age. As it was expected, our findings reveal that older women had more comorbidities, a higher ASA score at diagnosis, and were more likely to be diagnosed at more advanced stages (FIGO III–IV). This higher rate of advanced stage diagnosis could be explained by the unspecific symptoms that characterize ovarian cancer being dismissed or attributed to another disease more often in older women, thus delaying the diagnosis [1].

Regarding treatment, our study showed that older women more frequently underwent neoadjuvant chemotherapy followed by interval or rescue surgery. This approach is considered when a patient is initially not suitable for primary surgery due to factors like tumor extension or patient characteristics, as neoadjuvant chemotherapy aims to reduce tumor extension or minimize the extent of surgical resection, enabling these patients to better tolerate the subsequent surgery [5]. Despite this, older patients were less likely to achieve complete cytoreduction. Nevertheless, it is important to note that there were no significant differences between age groups when considering surgery complications. Hence, it could be inferred that if older patients were selected for cytoreductive surgery based on their general health status, they could fare as well as younger patients when it comes to surgery complications. Other studies support this theory, suggesting that underlying comorbidities rather than age alone are responsible for increased surgical morbidity and mortality in older patients [9]. On the other hand, it could also be suggested that surgery approach in older patients is less radical, which could lead to less surgical complications, even though the Aletti score showed no significant differences between age groups.

Lastly, the final aim of our study was to evaluate the impact of age and oncological management in survival. The study showed that despite older patients being diagnosed at later stages and undergoing more non-standard treatment, there were no differences in DFS between age groups. This could be explained by the natural history and progression of the disease and the fact that recurrence generally occurs at a high rate, especially between the first 12 to 24 months [20]. On the other hand, OS was lower in women aged 70 or older, possibly due to older patients receiving non-standard treatment more frequently and having a lower rate of complete cytoreduction. Both of these factors are known to impact prognosis according to published literature, although treatment type was not shown to be independent in our multivariate analysis [21,22].

The study does present some limitations. Most notably, it being a non-randomized control trial study, with common biases of observational studies. Another limitation is the sample size, which was relatively small, which limits the generalizability of findings and could impair the results, especially in the case of multivariate analysis. However, the patients included were treated by the same multidisciplinary team at the Hospital del Mar, following the same internationally accepted treatment guidelines for ovarian cancer. Secondly, as it consisted of a retrospective study, it is prone to selection and information bias, as well, data collection could have been conditioned. In fact, some variables were not available for every patient, and to reduce heterogeneity, some data were pooled into categories. This could affect the results when considering alternative explanations. As strengths, this study enhances the current understanding of ovarian cancer management in elderly patients, contributing to existing knowledge, and may help optimize treatment strategy in the future, potentially leading to improved patient outcomes.

## 5. Conclusions

In conclusion, considering the results of our study, we can affirm that women 70 or older are more likely to be diagnosed at later stages and treated less radically than younger women, with no age specific differences for DFS. Moreover, even though our results showed that women 70 or older had more comorbidities and a higher ASA classification at the time of diagnosis, it is important to consider that this only reflects a tendency in the group. Therefore, as age did not show to be an independent factor for DFS, the choice of treatment should not be based on age only, but should be individualized and consider the overall state of the patient, in order to achieve the best outcome possible when considering patient characteristics.

To expand knowledge on the matter and confirm our findings, future research should include larger multicentric prospective studies. Moreover, it would be ideal to create a guideline in order to select older patients suited for standard treatment better, instead of basing the decision mainly on age, and to finally be able to provide individualized treatment, achieving the best outcome considering patient characteristics.

## Figures and Tables

**Table 1:** Descriptive characteristics of ovarian cancer patients according to diagnostic age.

	Total Population (*n* = 110)	<70 (*n* = 73)	≥70 (*n* = 37)	*p*-Value	*N*
Age (years), mean ± SD	62.9 (±14.03)	55.2 (±10.2)	78.2 (±5.3)	NS	
BMI (kg/m^2^), median [range]	26.2 [23.8;30.8]	25.6 [23.1;31.2]	26.6 [25.4;29.4]	NS	110
Waist/hips ratio, median [range]	0.92 [0.85;0.99]	0.90 [0.83;0.98]	0.98 [0.92;1.00]	0.026	61
Smoker, *n* (%)				0.033	94
No	80 (85.1)	51 (79.7)	29 (96.7)		
Yes	14 (14.9)	13 (20.3)	1 (3.3)		
Menopause, *n* (%)				<0.001	110
No	24 (21.8)	24 (32.9)	0 (0.0)		
Yes	86 (78.2)	49 (67.1)	37 (100)		
Comorbidity Charlson Index, median [range]	4.00 [3.0;5.0]	3.00 [2.0;4.0]	6.00 [5.0;7.0]	<0.001	110
BRCA1/2 Mutation, *n* (%)				NS	92
No	74 (80.4)	49 (76.6)	25 (89.3)		
Yes	18 (19.6)	15 (23.4)	3 (10.7)		
Endometriosis, *n* (%)				NS	109
No	103 (94.5)	68 (93.2)	35 (97.2)		
Yes	6 (5.50)	5 (6.8)	1 (2.8)		
Albumin at diagnosis, median [range]	4.20 [3.9;4.6]	4.30 [4.0;4.6]	4.00 [3.6;4.5]	0.022	94
Hb at diagnosis, median [range]	12.9 [11.7;13.8]	13.1 [12.0;13.9]	12.3 [11.1;13.6]	NS	97
ASA ^1^, *n* (%)				0.013	110
1	4 (3.6)	3 (4.1)	1 (2.7)		
2	66 (60.0)	50 (68.5)	16 (43.3)		
3	38 (34.5)	20 (27.4)	18 (48.6)		
4	2 (1.9)	0 (0.00)	2 (5.4)		
Histological type, *n* (%)				NS	110
High-grade serous	72 (65.4)	46 (63.0)	26 (70.3)		
High-grade endometrioid	4 (3.6)	3 (4.1)	1 (2.7)		
Low-grade serous	7 (6.4)	4 (5.5)	3 (8.1)		
Low-grade endometrioid	7 (6.4)	6 (8.2)	1 (2.7)		
Clear cell	10 (9.0)	7 (9.6)	3 (8.1)		
Mucinous	5 (4.6)	3 (4.1)	2 (5.4)		
Others or undetermined	5 (4.6)	4 (5.5)	1 (2.7)		
Tumoral ascites, *n* (%)				NS	108
No	66 (61.1)	48 (66.7)	18 (50.0)		
Yes	42 (38.9)	24 (33.3)	18 (50.0)		
FIGO Stage at diagnosis ^2^, *n* (%)				0.012	109
I	29 (26.6)	24 (32.9)	5 (13.9)		
II	5 (4.6)	3 (4.1)	2 (5.6)		
III	56 (51.4)	30 (41.1)	26 (72.2)		
IV	19 (17.4)	16 (21.9)	3 (8.3)		

^1^ American Society of Anesthesiologists Classification; ^2^ FIGO: International Federation of Gynecology and Obstetrics; NS: non-significant.

**Table 2:** Treatment characteristics and outcomes of ovarian cancer patients included in the study.

	Total Population (*n* = 110)	<70 (*n* = 73)	≥70 (*n* = 37)	*p*-Value	*N*
Surgery type, *n* (%)				0.053	103
Primary	68 (66.0)	52 (73.3)	16 (50.0)		
Interval	24 (23.3)	14 (19.7)	10 (31.2)		
Rescue	11 (10.7)	5 (7)	6 (18.8)		
Aletti Score ^1^, *n* (%)				NS	100
≤3	35 (35.0)	20 (29.4)	15 (46.9)		
4–7	55 (55.0)	41 (60.3)	14 (43.8)		
≥8	10 (10.0)	7 (10.3)	3 (9.3)		
Duration of surgery (min), median [range]	270 [240;360]	300 [240;374]	270 [210;360]	NS	96
Clavien Dindo Classification, *n* (%)				NS	97
0	49 (50.5)	38 (56.7)	11 (36.6)		
1	5 (5.2)	3 (4.5)	2 (6.7)		
2	39 (40.2)	24 (35.8)	15 (50.0)		
3	4 (4.1)	2 (3.0)	2 (6.7)		
Surgery Outcomes, *n* (%)				0.024	103
Complete cytoreduction	94 (91.3)	68 (95.8)	26 (81.3)		
Incomplete cytoreduction	9 (8.7)	3 (4.2)	6 (18.7)		
Chemotherapy, *n* (%)				NS	110
no	22 (20.0)	14 (19.2)	8 (21.6)		
yes	88 (80.0)	59 (80.8)	29 (78.4)		
Chemotherapy cycles, *n* (%)				NS	88
1–3	4 (4.5)	1 (1.7)	3 (10.3)		
>3	84 (95.5)	58 (98.3)	26 (89.7)		
Chemotherapy complications grade, *n* (%)				NS	87
≤3	32 (36.8)	21 (36.8)	11 (36.7)		
>3	55 (63.2)	36 (63.2)	19 (63.3)		
Maintenance treatment, *n* (%)				NS	110
No	53 (48.2)	32 (43.8)	21 (56.8)		
Yes	57 (51.8)	41 (56.2)	16 (43.2)		
Treatment type, *n* (%)				0.023	110
Standard	89 (80.9)	64 (87.7)	25 (67.7)		
Non-standard	21 (19.1)	9 (12.3)	12 (32.4)		
Follow-up duration (months) median [range]	21.6 [13.8;32.5]	21.6 [13.8;33.1]	19.5 [13.8;27.7]	NS	110
Recurrence, *n* (%)				0.054	96
No	42 (43.7)	34 (50.0)	8 (28.6)		
Yes	54 (56.3)	34 (50.0)	20 (71.4)		
Death, *n* (%)				0.006	110
No	75 (68.2)	56 (76.7)	19 (51.4%)		
Yes	35 (32.8)	17 (23.3)	18 (48.6%)		

^1^ Aletti Surgical Complexity Scoring System; NS: non-significant.

**Table 3:** DFS. Univariate and multivariate analysis results.

	Univariate		Multivariate	
Variables	HR (95% CI)	*p*-Value	aHR (95% CI)	*p*-Value
Age				
<70	1		1	
≥70	1.67 (0.95–2.91)	NS	1.24 (0.55–2.78)	NS
Comorbidity Charlson Index	1.26 (1.03–1.41)	0.021	1.16 (0.9–1.5)	NS
FIGO stage at diagnosis				
I–II	1		1	
III–IV	4.58 (2.14–9.81)	<0.001	3.44 (1.5–7.87)	0.003
Tumoral ascites				
No	1		1	
Yes	3.24 (1.81–5.79)	<0.001	2.23 (1.15–4.35)	0.018
Treatment type				
Standard	1		1	
Non-standard	1.42 (0.63–3.16)	NS	0.58 (0.24–1.4)	NS

aHR: adjusted hazard ratio; NS: non-significant.

**Table 4:** OS. Univariate and multivariate analysis results.

	Univariate		Multivariate	
Variables	HR (95% CI)	*p*-Value	aHR (95% CI)	*p*-Value
Age				
<70	1		1	
≥70	3.11 (1.58–6.12)	0.001	1.74 (0.61–4.98)	NS
Comorbidity Charlson Index	1.47 (1.23–1.76)	<0.001	1.25 (0.93–1.69)	NS
FIGO stage at diagnosis				
I–II	1		1	
III–IV	3.11 (1.10–8.83)	0.033	1.84 (0.56–5.99)	NS
Tumoral ascites				
No	1		1	
Yes	2.88 (1.43–5.79)	0.003	1.65 (0.70–3.87)	NS
Treatment type				
Standard	1		1	
Non-standard	3.24 (1.52–6.92)	0.002	1.44 (0.58–3.54)	NS

aHR: adjusted hazard ratio; NS: non-significant.

## Data Availability

The data presented in this study are available in this article.

## Abbreviations

The following abbreviations are used in this manuscript:

OS	overall survival
HR	hazard ratio
DFS	disease-free survival
SAMIUC	Sociedad Andaluza de Medicina Intensiva y Unidades Coronarias
FIGO	International Federation of Gynecology and Obstetrics
SD	standard deviation
aHR	average hazard ratio
ASA	American Society of Anesthesiologists
